# Clinicians risk becoming ‘liability sinks’ for artificial intelligence

**DOI:** 10.1016/j.fhj.2024.100007

**Published:** 2024-02-19

**Authors:** Tom Lawton, Phillip Morgan, Zoe Porter, Shireen Hickey, Alice Cunningham, Nathan Hughes, Ioanna Iacovides, Yan Jia, Vishal Sharma, Ibrahim Habli

**Affiliations:** a Improvement Academy, Bradford Institute for Health Research, Bradford Royal Infirmary, Duckworth Lane, Bradford BD9 6RJ, UK; b Assuring Autonomy International Programme, University of York, Heslington, York YO10 5DD, UK; c York Law School, University of York, Heslington, York YO10 5DD, UK; d Department of Computer Science, University of York, Heslington, York YO10 5DD, UK

## The problem

Artificial Intelligence (AI) is often touted as healthcare's saviour, but its potential will only be realised if developers and providers consider the whole clinical context and AI's place within it. One of many aspects of that clinical context is the question of liability.

Analysis of responsibility attributions in complex, partly automated socio-technical systems has identified the risk that the nearest human operator may bear the brunt of responsibility for overall system malfunctions.^1^ As we move towards integrating AI into healthcare systems, it is important to ensure that this does not translate into clinicians unfairly absorbing legal liability for errors and adverse outcomes over which they have limited control.

**Fig. 1:**
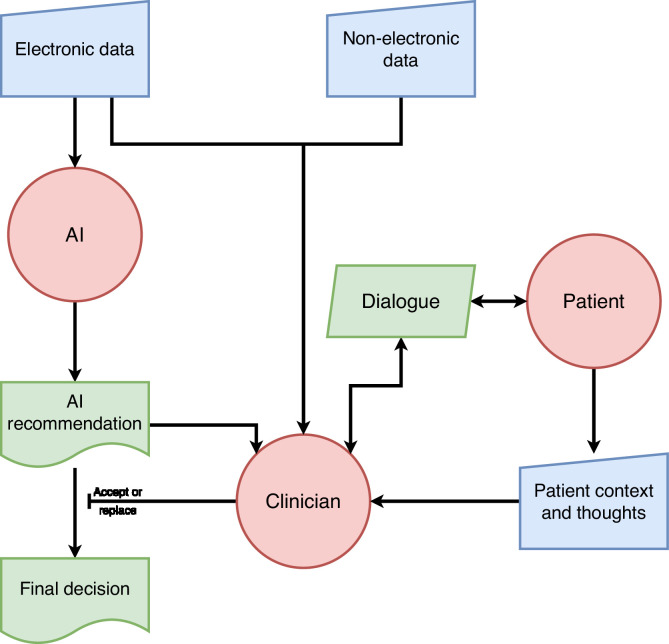
Current prevalent AI model.

In the current, standard model of AI-supported decision-making in healthcare, electronic data is fed into an algorithm, typically a machine-learnt model, which integrates the acquired information to arrive at a recommendation which is output to a human clinician. The clinician can consider this recommendation alongside information from other sources, including examination of and discussion with the patient, and either accept the recommendation as-is, or replace it with a decision they make themselves (Fig. 1). For example, in a system recommending treatment for diabetes, the system may recommend – based on coded electronic data – that it is appropriate to start insulin; though after considering patient context and wishes the clinician may choose to override this. Due to differences in regulatory approval processes, the positioning of such systems as clinical support rather than diagnostic makes them cheaper and quicker to get to market. Additionally, given recent guidance from the National Health Service in England, which clarifies that the final decision must be taken by a healthcare professional,^2^ this model looks set to become the norm across the UK healthcare system.

But the standard model may have a negative impact on the clinician, who must choose between accepting the AI recommendation, or substituting their own decision - which, despite probably being AI-influenced, involves largely reverting to a traditional (non-AI) approach. They risk no longer doing what they are best at, including exercising sensitivity to patient preferences and context, but in effect acting as a sense-check on, or conduit for, the machine. There has been substantial discussion of the cognitive and practical challenges humans face when monitoring automation, such as the additional load of maintaining effective oversight, ensuring sufficient understanding to identify a fault in the system, changes to the way they evaluate information sources, and automation bias.^3^^,^^4^ For instance the clinician may lack knowledge about the training dataset of the diabetes recommendation system and be unaware that it is less accurate for patients from some ethnic backgrounds; meanwhile, its influence may make the clinician more likely to question their own evaluation. At the same time, the guidance states that the clinician may be held legally accountable for a decision made using the support of AI.^2^ Analogous to the way a ‘heat sink’ takes up unwanted heat from a system, the human clinician risks being used here as a ‘liability sink’, where they absorb liability for the consequences of the AI's recommendation whilst being disenfranchised from its decision-making process, and also having difficult new demands placed on them.

A similar situation exists in driver assistance and self-driving systems for cars, where despite the AI being in direct control of the vehicle, in some jurisdictions it seems the human in the driving seat is already being used as a liability sink. For example, a driver activating self-driving mode typically has to accept that they will take over manual control immediately when required. But in many Tesla collisions Autopilot aborted control less than one second prior to the first impact.^5^ This does not give the driver enough time to resume control safely - and yet in practice, for jurisdictions that adopt fault based systems of liability for motor vehicle accidents such the UK, it is likely that they would be liable for the accident. As the most obvious ‘driver’ close to where AI is used in a clinical setting, the clinician could easily end up being held similarly liable for harmful outcomes from AI-based decision-support systems, and carrying this stress and worry, but having limited practical control over their development and deployment, or understanding of how the AI recommendations are reached.^6^

Besides becoming liability sinks for AI, many clinicians will undoubtedly take on personal accountability for adverse consequences. Clinicians involved in patient safety incidents or errors in health care often feel responsible,^7^ even when this responsibility lays within the organisation or system in which they work. Clinicians can become ‘second victims’ and suffer serious mental health consequences after such an incident, including depression, anxiety, PTSD and even suicide.^8^ Not all the after-effects of being involved in a patient safety incident are negative. Clinicians often learn in the aftermath of an incident, prompting constructive change which promotes patient safety and prevents future similar incidents.^9^ But how can we learn after a patient safety incident for which an AI is responsible, when we do not understand how that decision or error was made? Without this positive aspect and coping mechanism of involvement in a patient safety incident, it is likely the second victim experience for many will be more significant, and the lesson will rapidly be to not trust or use the AI.

## Possible solutions

The attribution of liability in a whole socio-technical system becomes complex when AI is involved. As well as the humans directly present at the event, there were humans involved in the design and commissioning of the AI system, humans who signed off on its safety, and humans overseeing its running or working in tandem with it. Complexity is further increased with AI because human oversight may be more influenced by automation bias - where humans attribute greater than warranted intelligence to the machine - and because the AI's decision-making cannot be clearly understood by its operators. Given that automation bias and AI inscrutability are problems across many settings where AI is used, it is no surprise that efforts are already being made to solve them.^10^^,^^11^

Whilst we are some way off it being possible, or even appropriate, to hold an AI system itself liable,^12^ any of the humans involved in an AI's design, building, provisioning, and operation might be held liable to a degree. Smith and Fotheringham argue that using clinicians as the sole focus for liability is not ‘fair, just and reasonable’.^13^ Without a clear understanding of how an AI came to a decision, a clinician is faced with either treating it as a knowledgeable colleague,^14^, ^15^, ^16^ or coming to their own judgement and largely ignoring the AI - or even turning it off. Even if they resolve to make their own decision and then check it against the AI's recommendations, this only avoids the problem when there is agreement. If the AI disagrees, the clinician faces the same dilemma.

Although the AI system is a product, product liability does not provide an attractive alternative for claimants, versus a claim against the clinician and their employer via vicarious liability. Product liability claims are notoriously difficult, and forensically expensive, compared with ordinary professional negligence claims. The claimant needs to identify and prove the defect in the product. Within the context of opaque and interconnected AI, this may be an extremely difficult and costly exercise requiring significant expertise.^17^^,^^18^ Also, the current product liability regime in England and Wales, found in the Consumer Protection Act 1987, based on the EU Product Liability Directive (PLD),^19^ predates the digital age. It has significant problems in an AI context. Software, when not delivered alongside hardware, appears not to be a ‘product’ for the purposes of this regime, and the assessment of defectiveness occurs at the time of the supply of the hardware, so over the air updates and post-delivery learning are not relevant. The regime also contains a state-of-the-art defence, strengthening the position of producers. Although the fault-based tort of negligence (based on the manufacturer's/producer's duty of care) may also be deployed in a product liability context, such claims also have significant problems in an AI context.^18^ Unfortunately, the clinician and their employer via vicarious liability for the clinician's negligence, remain the most attractive defendants to sue.^20^

‘Vicarious liability’ is when an employer is held strictly liable for the negligence or wrongdoing of an employee, which is closely connected to their employment. The wrongdoing of the employee must be established first. In a medical negligence context, negligence still traditionally focuses on the individual, with the hospital being vicariously liable for that individual's tort – although a system-based model would perhaps be better, both for patient safety and for the impact on individual clinicians.^21^ Even if the clinician's employer ultimately pays, this vicarious liability is based on a finding that the clinician themselves is at fault.

There may be alternative claims against the hospital (which also owes a duty of care to the patient) for systemic negligence, for instance regarding the staff training on such technologies. At a stretch a claimant could attempt to target regulators, such as the Care Quality Commission (CQC), although establishing their duty of care to the relevant claimant will be a substantial hurdle to cross. The negligence claim against the clinician is easier to establish, requires less evidence, and in particular does not require extensive proof of causation, unlike actors further up the causal chain. The doctor at the chain's end is thus a softer target. Piggy-backing their employer via vicarious liability ensures a solvent defendant.

Meanwhile, AI systems are currently treated as products, so the software development company (SDC) would only be liable to the patient through product liability, which we have seen above has considerable problems in this context. In the future, it may be that the AI system is treated as part of the clinical team – and not as a product – so that its ‘conduct’ could be attributed to those who ‘employ’ the AI system, which may for instance be the SDC, or clinician's trust.^18^ But that is not the current legal context. It is also unclear what ‘standard of care’ would apply to an AI that is treated as part of the clinical team: that of the reasonable AI system, or that of the reasonable clinician?^22^ The SDC might argue that the higher standard is unreasonable. But this implies that their system is simply not good enough - that its recommendations are inferior to the decisions of a clinician - and few organisations would be willing to deploy an AI system on that basis.

Smith and Fotheringham argue that there should be risk pooling between clinicians and SDCs for harms - with actuarially-based risk pooling insurance schemes to provide cover for AI-related damage.^13^ However, these are at present merely proposals. Currently, a clinician (using an AI system) who is held liable in negligence to the patient may seek contribution from the SDC via the Civil Liability (Contribution) Act 1978, although, as with the patient's claim against the SDC there are significant difficulties in doing so, since as noted above establishing that the SDC is itself liable for the damage suffered is problematic. The SDC may also have sought to contractually exclude any right of clinicians to seek such contribution. Thus, in practical terms with systems of this type the clinician remains liable for acting on the recommendations or decisions of an AI they do not and cannot fully understand. Facing the stress and worry of the consequences of using it, many clinicians may refuse to accept the risk, and simply turn off the machine.

## Alternative models

Pooling risk might prevent the clinician becoming a liability sink, but the prevalent model may have other drawbacks for the clinician, the patient, and the system as a whole. Fig. 1 shows that the entire input of the patient and clinician into the decision is restricted to either accepting the AI's recommendation, or - for this case - rejecting it (effectively switching it off and returning to standard practice, albeit likely influenced by the AI's recommendation). This is at odds with the goal of patient-centred decision-making,^23^ as the AI cannot easily incorporate patient context and ideas, concerns, and expectations itself - this context is only added by the clinician choosing to accept or replace the AI's output. It may also be frustrating for the clinician by eroding their ability to do what they do best: integrating clinical science and patient context in a dialogue to come to a shared decision.

**Fig. 2:**
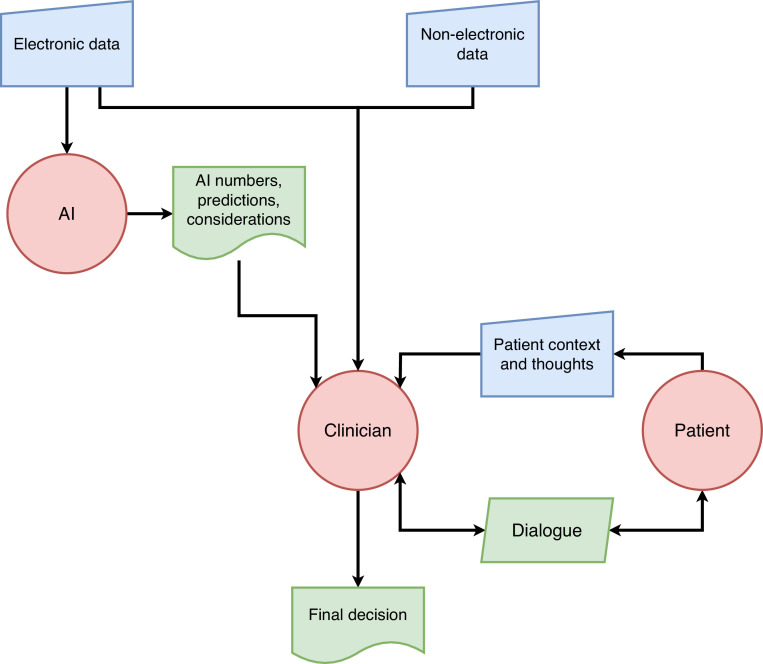
AI model with alternative outputs to inform patient/clinician dialogue.

Fortunately, this is not the only possible approach. Rather than restructuring systems in a clinical setting around an AI designed to work this way, it may be preferable to explore alternative models which give greater focus to the patient and clinician.^24^ In some of these models the AI may not even give a decision or recommendation, but instead show predictions of the effect of different decisions (e.g. treatment options), or highlight data that is most relevant to the AI model in its decision making. In this way, the explanation of an explainable-AI system may be more useful than the decision or recommendation itself.^25^^,^^26^ Fig. 2 shows a model where these alternative outputs from the AI system inform a dialogue between the clinician and patient, leading to a decision. Whilst the model refers to complex AI systems which cannot be directly interpreted by anyone, including the clinician, this is clearly analogous to existent non-AI systems such as automated ECG analysis where the inner workings are not easily available to the clinician.

In the diabetes example, the outputs may be predictions for each treatment option of outcomes such as blood test results or other endpoints such as the risk of a heart attack, forming the basis for the dialogue and subsequent decision, and the AI may not output a direct treatment recommendation at all. Most current AI radiology systems are similar – providing information to a reporting clinician to highlight areas and possible diagnoses without directly completing the report. There are vendors attempting to take the clinician out of the loop, but presently systems can only take on a small proportion of the workload.^27^ As these truly autonomous systems advance, without a nearby clinician liability sink, they may well test some of the legal issues discussed above.

**Fig. 3:**
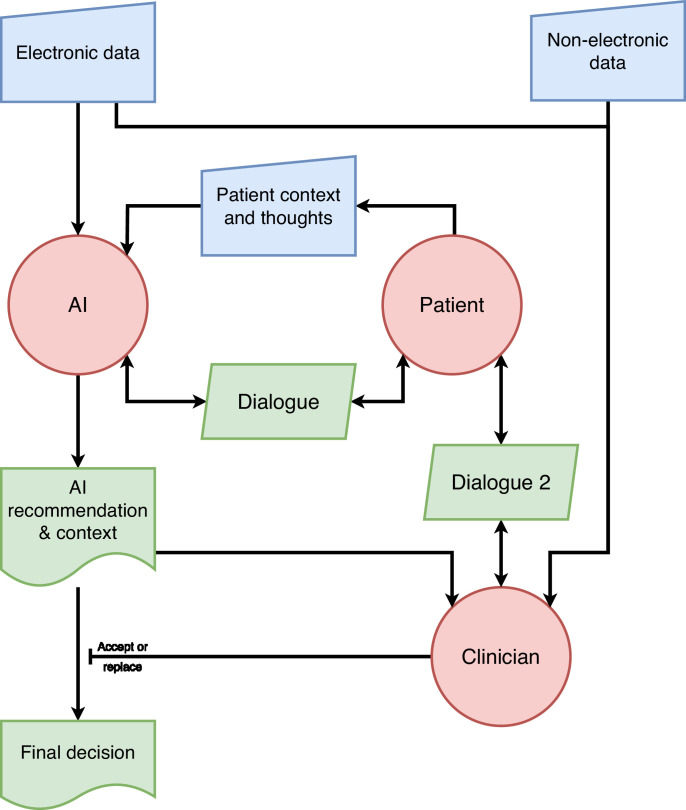
Advanced AI model capable of sustaining dialogue with the patient.

In Fig. 3, a more advanced AI system communicates directly with the patient and a three-way dialogue proceeds before a decision emerges. A year ago, dialogue with an AI capable of explaining itself to patients might have been considered fanciful, but advances in Large Language Models employed in tools like ChatGPT have made them seem very plausible. A diabetes system built this way might be capable of eliciting the patient's thoughts and concerns about the difficulties of starting insulin. It could provide a tailored approach that does not lose the patient voice, and provide an explanation to the clinician in more the manner of discussion with a multidisciplinary team member. Other models can be conceived along these lines, bringing the patient and clinician back into the decision-making focus.

With both models in Figs. 2 and 3, the clinician retains the final decision as recommended by NHS England. Are they still a liability sink for the AI? The models may not remove liability, but we would argue that the clinician's role here is much more traditional, as they are integrating a variety of data and opinions in a manner of working that has become familiar with the advent of the multidisciplinary team.^28^ Clinicians should feel much more comfortable in accepting liability for a decision where they have genuine understanding and agency, and the socio-technical system as a whole will be much more acceptable to both clinicians and patients as it retains compatibility with patient-centred care.

The question remaining in this setup, however, is the assignment of liability for defective AI advice or information. As these models return the clinician to a more traditional role, the current legal position becomes more appropriate: treating the AI as a standard medical device. This could be dealt with via product liability, suitably adjusted to take into account the problems within such regimes as applied to AI systems, such as proof of causation, and the failure discussed above of the PLD^19^ to cover unembodied software. The European Union has recognised this need, and published reform proposals for the PLD. If we do not want clinicians to become liability sinks, similar reforms may be needed in the United Kingdom.

In summary, AI systems being developed using current models risk using clinicians as ‘liability sinks’, absorbing liability which could otherwise be shared across all those involved in the design, institution, running, and use of the system. Alternative models can return the patient to the centre of decision-making, and also allow the clinician to do what they are best at, rather than simply acting as a final check on a machine.

## Summary

• The benefits of AI in healthcare will only be realised if we consider the whole clinical context and the AI's role in it.
• The current, standard model of AI-supported decision-making in healthcare risks reducing the clinician's role to a mere ‘sense check’ on the AI, whilst at the same time leaving them to be held legally accountable for decisions made using AI.
• This model means that clinicians risk becoming ‘liability sinks’, unfairly absorbing liability for the consequences of an AI's recommendation without having sufficient understanding or practical control over how those recommendations were reached.
• Furthermore, this could have an impact on the ‘second victim’ experience of clinicians.
• It also means that clinicians are less able to do what they are best at, specifically exercising sensitivity to patient preferences in a shared clinician-patient decision-making process.
• There are alternatives to this model that can have a more positive impact on clinicians and patients alike.

## CRediT authorship contribution statement

**Tom Lawton:** Conceptualization, Funding acquisition, Writing – original draft, Writing – review & editing, Formal analysis, Visualization. **Phillip Morgan:** Writing – original draft, Formal analysis, Visualization, Writing – review & editing. **Zoe Porter:** Conceptualization, Funding acquisition, Writing – original draft, Writing – review & editing, Formal analysis, Visualization. **Shireen Hickey:** Writing – original draft, Formal analysis, Visualization, Writing – review & editing. **Alice Cunningham:** Formal analysis, Visualization, Writing – review & editing. **Nathan Hughes:** Formal analysis, Visualization, Writing – review & editing. **Ioanna Iacovides:** Formal analysis, Visualization, Writing – review & editing. **Yan Jia:** Formal analysis, Visualization, Writing – review & editing. **Vishal Sharma:** Formal analysis, Visualization, Writing – review & editing. **Ibrahim Habli:** Conceptualization, Funding acquisition, Writing – original draft, Writing – review & editing, Formal analysis, Visualization.

## Declaration of competing interest

TL has received an honorarium for a lecture on this topic from Al Sultan United Medical Co and is head of clinical artificial intelligence at Bradford Teaching Hospitals NHS Foundation Trust, and a potential liability sink

All other authors report no conflicts of interest
